# 8–11 year results of LARS augmented hamstring in anterior cruciate ligament reconstruction surgery: Incidence of synovitis and failures

**DOI:** 10.1016/j.jor.2025.06.033

**Published:** 2025-07-01

**Authors:** Sol Qurashi, Wagdy Ashaia, Janna Maier, William Ridley, Tat Chao, Muhaimen Jassim, Sam Aktas, Femi E. Ayeni, Raj Narulla

**Affiliations:** a Dept of Orthopaedics, Nepean Hospital, Kingswood NSW 2747, Australia; b The Hip and Knee Clinic Penrith NSW, 2750, Australia; c Canterbury Hospital, Campsie NSW 2194, Australia; d Hawkesbury Hospital, Windsor NSW, 2756, Australia; e Nepean Institute of Academic Surgery, Nepean Clinical School, The University of Sydney. Australia; f University of Notre Dame, Chippendale NSW, 2007, Australia

**Keywords:** Hamstring, Anterior cruciate ligament, Ligament augmentation and reconstruction system, Synovitis, Autograft

## Abstract

**Background:**

Anterior cruciate ligament (ACL) injury account for the majority of sport related knee ligament injuries and ACL reconstruction surgery is one of the most performed operations in the young adult population. Various graft types have been used to reconstruct the ACL, however there is no consensus on the best option. Concerns about quality and size of the graft and its effects on success of the reconstruction remain. Synthetic grafts have also been used for a long time as a substitute or to augment biological grafts however, concerns about early failure and particle related synovitis have limited their intra-articular use. The most common synthetic graft option still in use is the Ligament Augmentation and Reconstruction System (LARS).

**Method:**

The study is a case controlled retrospective comparison of 102 consecutive cases reporting 8–11 year results in relation to clinical outcomes revision rates and synovitis of ACL reconstruction surgery using LARS synthetic grafts as an augment when compared with hamstring graft alone.

**Result:**

The study found a mean follow up was 9 years and one month (8–11 years) and revision rate was 5.7 % for the LARS augmented group and 12.5 % for the Hamstring group, but not significantly different, odds ratio 0.4 (C.I: 0.1179–1.555, p = 0.255). LARS augmented autograft ACL reconstruction had similar results at 8–11 years when compared to autograft only reconstructions. No synovitis was noted in either group.

**Conclusion:**

LARS ensures an appropriately sized graft, is safe and not associated with synovitis in our cohort at 8–11 year follow up.

## 1 Introduction

Surgical management of Anterior Cruciate Ligament (ACL) ruptures is a complex and evolving field. The ACL is an important stabiliser of the human knee, resisting both anterior tibial translation and internal tibial rotation.^1^ Data from the Mayo clinic estimates the incidence of ACL tears by around 68.6 per 100,000 person-years, most commonly in the age group of 14–25 years with a male preponderance.^2^ Controversy exists surrounding surgical decision making in the ACL injured patient, particularly so with ACL graft choice. Graft options include autograft, allograft and synthetic grafts.^3^

Graft size in hamstring autograft ACL surgery is an important factor directly related to failure. The majority of the evidence on the topic suggests that the size of the graft in hamstring, by far the most common, autograft ACL reconstruction matters in avoiding failures. Multiple studies have shown a strong correlation between graft size and failure risk including a meta-analysis quantifying this at a 6.8 times greater relative risk of failure for grafts ⩽ 8 mm in diameter (p = 0.008). This literature suggests a graft of at least 8.5-mm to decrease the risk of having a failure.^4^, ^5^, ^6^

Various strategies can be used to achieve a bulkier graft. These include increasing stranding in autograft as well as the use of synthetic augmentation devices. The most well-known of these is the Ligament Augmentation and Reinforcement System, also known as the LARS device. LARS is one of the most commonly used synthetic grafts for ACL reconstruction. It is a non-absorbable material made of terephthalic polyethylene polyester which is machined to decrease the risk of tissue reaction and encourage tissue in-growth.^7^ LARS can be used as a standalone graft for ACL reconstruction or as in augmentation of auto or allograft.^8^ LARS enjoyed early popularity when first released, but utilisation had since reduced due to case reports of LARS associated synovitis and associated failures.^9^, ^10^, ^11^, ^12^, ^13^ These concerns led to a reluctance in the orthopaedic community to use LARS for ACL reconstruction even though the concerns were not well founded with high level evidence.

On the contrary, a recently published meta-analysis demonstrated some benefit with the use of LARS, concluding that reconstruction using LARS resulted in better postoperative outcomes regarding knee joint function and stability compared to autografts.^14^

In this context, the study aims to assess the safety of LARS augment, specifically regarding the previously raised concerns of incidence of synovitis and early failure when compared to a 4-strand hamstring and report 8–11year results of the two cohorts.

## 2 Methods

**Fig. 1:**
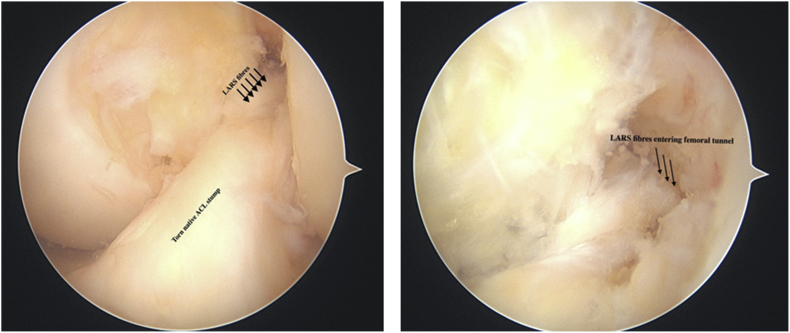
LARS augment through existing ACL stump (left) and close up image of fibres (right).

We retrospectively analysed prospectively collected demographic and functional data for 102 consecutive patients from December 2012 to March 2017 who underwent elective ACL reconstruction by a single Surgeon. All patients had a general anaesthetic, tourniquet, and knee arthroscopy utilising standard portals. Meniscal repair or debridement was performed for meniscal pathology as needed. A longitudinal incision was made over pes anserinus for harvesting of hamstrings graft on the ipsilateral knee leaving no attached ends of the hamstring graft. The graft was then measured and if the four strands hamstring graft thickness was less than 7.5 mm, a LARS augment was added (Fig. 1). The graft (hamstrings or combined hamstrings and LARS) was secured with an Endobutton (Smith and Nephew) on the femoral side and a non-absorbable Biosure Peek interference screw and reinforced with a small staple (Smith and Nephew) on the tibial side. Graft type, size and femoral tunnel size were all recorded. All patients had a standard ACL physiotherapy protocol which allowed for immediate weight bearing and closed chain exercises for the first three months post-operatively and no cross cutting, sudden turning or pivoting activities or return to sport for 10 months. This did not vary between the autograft or LARS augmented groups. Patients were followed-up at two weeks, six weeks and three months, with an additional phone-call at the time of study finalisation to check for complications, functional status in terms of exercise and employment and revisions. Tegner Lysholm score was also collected. In the absence of a reliable non-invasive test for synovitis, we interpreted the pain and swelling components of the Tegner Lysholm Score as surrogates for synovial change.

### 2.1 Statistical analysis

Univariate descriptive statistics was performed on extracted data as required. Continuous variables were recorded as a mean and range and categorical variables as a percentage. Comparisons between quantitative data were performed using the *t*-test, and categorical data using the Fisher's exact test. and followed by logistic regression analyses of the outcome variables. For all analyses, P-values of less than 0.05 was considered statistically significant.

## 3 Results

Between December 2012 and March 2017, 105 ACL reconstructions were performed by a single surgeon. 102 of these were contactable. 70 had a LARS augmented Hamstring autograft and 32 had a hamstrings autograft only. There was a total of 33 females and 69 males in the study. There was no significant difference in gender distribution between the two operative groups (p = 0.335). Patients who had hamstring autografts were significantly younger (mean age 27 years) than patients who had LARS augmented Hamstring autograft (mean age 33.07 years) (p = 0.008). Mean follow up was 9 years and one month (8–11 years). Mean femoral tunnel size, for both groups, was 8 mm. There was no difference in the number of in additional procedures performed (meniscal repair or debridement) between the two groups, P > 0.05.

**Fig. 2:**
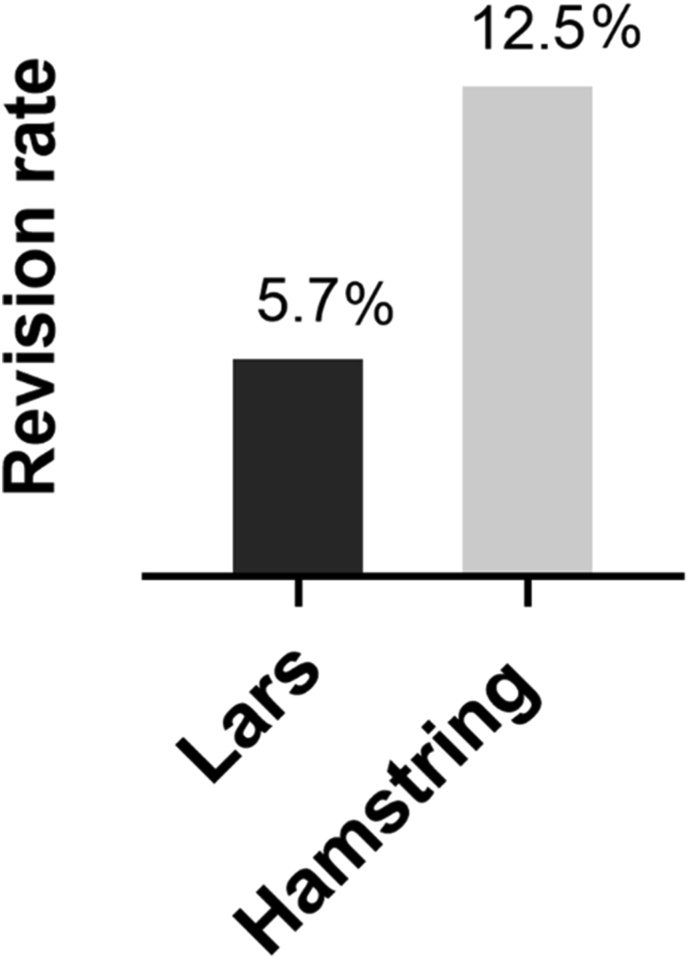
Revision rate of LARS augment v/s hamstring alone.

There were 14 repeat procedures; 2 arthroscopies for meniscal pathology, one removal of prominent staple, one of endobutton failure changed to xtendobutton but without graft revision, 2 total knee replacements (TKR) for progression of arthritis without ACL instability at time of TKR and 8 revision ACL procedures for failure of ACL reconstruction and ACL related instability. The revision rate for ACL failure was 5.7 % (n = 4/70) for the LARS augmented group and 12.5 % (n = 4/32) for the Hamstring group (Fig. 2). Failure rate was not affected by age, sex, graft type or size on regression analysis, P > 0.05. All ACL revisions were performed for repeat injuries after return to full sport (1, 2, 4, 5 and 10 years) post index procedure. No synovitis was noted in any of these, and no cases of synovitis were seen in any of the other patients.

**Fig. 3:**
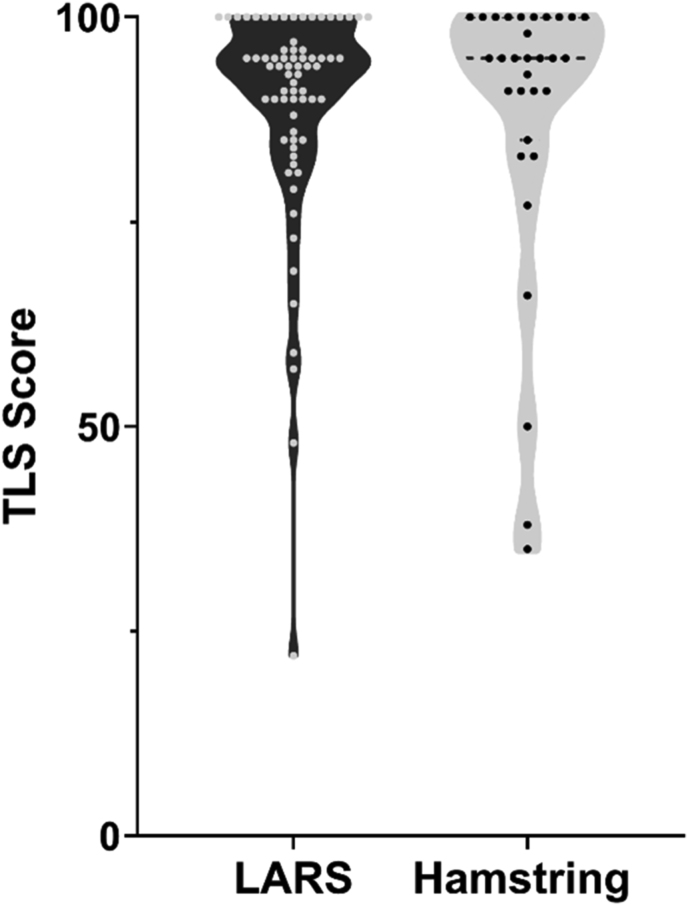
Tegner Lysholm scores.

The mean of Tegner Lysholm (TL) score was 89.47 (95 % CI; 86.2–92.7) for the LARS augmented group and 88.29 (95 % CI; 81.2–94.8) for the hamstring group and not significantly different, p = 0.7418 (Fig. 3). Constant swelling but not constant pain was observed in only one patient who had a LARS augmented graft. This patient had returned to full sport. Constant pain was observed in three patients, two of whom had a LARS augmented graft and one of whom had hamstring graft. None of these patients had constant swelling and all were back to usual occupation and sport. Two patients had instability symptoms, one with a LARS augmented graft, and one with a hamstrings graft.

Rate of return to their preinjury activity of work and/or sport was higher in the LARS group when compared to the Hamstring only group, odds ratio 2.4 (92 % versus 84 %), but was not significantly different (CI: 0.6955–8.231, p = 0.2797).

## 4 Discussion

Synthetic grafts have the advantages of coming in different sizes and lengths. Their use also whilst potentially avoids the potential donor site morbidity associated with autografts and the infection risk concerns with the use of allograft. Synthetic grafts can be used as an augment to autograft or as the sole graft for ACL reconstruction.^15^ In our series, the LARS ligament was used as an augment, paired with hamstring graft and used in situations where the autograft diameter would have otherwise been 7.5 mm or less. As a result, in our series there was no graft, autograft alone or LARS augmented autograft, that had a diameter of less than 7.5 mm. The use of LARS therefore ensured a minimum diameter of graft, and this in turn ensures greater graft strength and a higher threshold to rupture as shown previously in multiple publications.^4^, ^5^, ^6^ Moreover, this was achieved without any incidence of synovitis as demonstrated by the absence of any statistically significant difference in the Tegner Lysholm Scores between the two groups.

Synthetic devices for ACL reconstruction were introduced as early as the 1970s but unacceptable failure rate and inflammatory reaction lead to early discontinuation of their use.^15^ Since that time, different materials and techniques have been applied.

In the year 2000, LARS came into clinical use with promising results reported by Lavoie et al..^16^ Further case series continued to show the safety and efficacy of the LARS graft. Parchi et al., reported on 26 ACL reconstructions using the LARS graft only with an average follow-up of 11.6 years. The study reported overall satisfactory results in 22 patients and four patients with mechanical failure. There were no cases of synovitis or infection.^17^ A systematic review by Newman et al., had equivocal results and called for further studies to determine superiority of LARS over autograft. The authors noted synovitis to be a rare complication related to improper graft position.^18^

Another systematic review by Mulford et al. noted good short-term results and faster recovery times with using LARS compared to autografts.^19^ Li et al., compared 24 ACL reconstructions using LARS to 26 ACL reconstructions using bone-patellar tendon-bone (BPTB) with a mean follow-up period of two years. The authors found earlier functional recovery in the LARS group.^20^

A multicentre study on the outcome of ACL reconstruction using LARS with 3–5 years follow up, found 94 % of patients were graded A or B according to the International Knee Documentation Score (IKDC) and 93 % of patients were very satisfied or satisfied. In this study, 3 patients out of 159 developed synovitis.^21^ Another study using LARS at7 years follow up found improvement in IKDC and Lyshlom score with 86.8 % of patients returned to sports. The failure rate was 4.4 % and the overall complication rate was 2.2 %.^22^ A study that investigated the use of LARS to augment short or undersized hamstring tendon graft, has found similar results and concluded that LARS is safe and satisfactory option for ACL reconstruction.^23^ There were few case reports published regarding the incidence of knee synovitis after using LARS for ACL reconstruction^10^ but this seemed to be the most common misconception used to justify avoiding the use of LARS amongst orthopaedic surgeons.

A systematic review of synthetic devices for ACL reconstruction by Batty et al., found that LARS had lower rate of failure, revision and synovitis compared to the older devices.^24^ In a multicentre study of 159 patients who had ACL reconstruction using LARS with mean follow-up of 50 months, 94 % of patients had IKDC (International Knee Documentation Committee Score) of A or B and 93 % of patients were satisfied or very satisfied with their outcomes. Only one patient developed knee synovitis, and three patients had graft rupture.^21^ A study by Ranger et al. to evaluate using LARS for multi-ligament reconstruction after Knee dislocation found favourable outcomes and sustained knee stability with a minimum follow-up of 2 years.^25^

Our results are comparable to the above review of the literature, with similar or better results for graft rupture, infection, revision, and patient reported outcome measures.

Constant pain and swelling together as a proxy for synovitis were not observed together in any patient in either of the groups. There were no revision cases that were due to constant pain or swelling. Revisions were due to graft rupture from repeat injuries and not synovitis in this series. The number of patients who returned to unrestricted sport or exercise were comparable to other previously published studies, and there was a higher proportion of patients in the LARS group who returned to full activities.^26^

Our series has demonstrated similarly good outcomes between patients who had their ACL reconstructed using a LARS augmented hamstring graft and those reconstructed with a hamstring graft. Our study limitations include the small sample size, influencing its power and the retrospective nature of the study. We also appreciate that a source of bias may arise from the graft/tunnel size as LARS augment were only used if the graft size was 7 mm or less. Even so, the use of LARS provides an opportunity to achieve a bigger graft size and do so safely when compared with harvesting contralateral hamstring or allograft which make it a practical option. Whilst acknowledging the above, given the lack of specific evidence in this topic, we feel that the duration of our follow-up enhances the relevance of the study and its impact.

## 5 Conclusion

Our results demonstrate that LARS is a viable augmentation option in ACL reconstruction, providing a safe, stable, well sized graft without evidence of synovitis. It yields similar results for patients when combined with undersized autologous grafts compared with those patients with larger autologous hamstring graft at 10 years. Specifically, in regard to synovitis, our study did not show the incidence of synovitis that is commonly quoted as being associated with the use of LARS.

## Guardian/patient's consent

Not applicable.

## Statement of ethics

This study was performed in line with the principles of the Declaration of Helsinki. Approval was granted by the Ethics Committee of Nepean Blue Mountains Local 10.13039/100018696Health District (NBMLHD) Human Research Ethics Committee (HREC) LNR/18/NEPEAN/17.

## Credit author statment

Q.S.: Conceptualization, Methodology, Resources, Data curation, Writing – original draft Preparation, Writing – review & editing. A.W.: Methodology, Formal analysis, Writing – original draft Preparation. J.M.: Methodology, Formal analysis, Writing – original draft Preparation. R.W.: Methodology, Data curation, Writing – original draft Preparation. C.T.: Methodology, Data curation, Writing – original draft Preparation. M.J.: Methodology, Data curation, Writing – original draft Preparation. A.S.: Writing – original draft Preparation. FEA: Formal analysis, Writing – review & editing. N.R.: Methodology, Writing – original draft Preparation. All authors read and approved the final manuscript.

## Funding statement

This research did not receive any specific grant from funding agencies in the public, commercial or not-for-profit sectors.
